# Shared and distinct voxel-based lesion-symptom mappings for spasticity and impaired movement in the hemiparetic upper limb

**DOI:** 10.1038/s41598-022-14359-8

**Published:** 2022-06-17

**Authors:** Silvi Frenkel-Toledo, Mindy F. Levin, Sigal Berman, Dario G. Liebermann, Melanie C. Baniña, John M. Solomon, Shay Ofir-Geva, Nachum Soroker

**Affiliations:** 1 Department of Physical Therapy, School of Health Sciences, Ariel University, Ariel, Israel; 2 Department of Neurological Rehabilitation, Loewenstein Rehabilitation Medical Center, Raanana, Israel; 3 School of Physical and Occupational Therapy, Faculty of Medicine and Health Sciences, McGill University, Montreal, Canada; 4 Center for Interdisciplinary Research in Rehabilitation (CRIR), Montreal, Canada; 5 Department of Industrial Engineering and Management, Ben-Gurion University of the Negev, Beer-Sheva, Israel; 6 Zlotowski Center, Ben-Gurion University of the Negev, Beer-Sheva, Israel; 7 Department of Physical Therapy, The Stanley Steyer School of Health Professions, Sackler Faculty of Medicine, Tel-Aviv University, Tel Aviv, Israel; 8 Department of Physiotherapy, Manipal College of Health Professions, Manipal Academy of Higher Education, Manipal, Karnataka India; 9 Centre for Comprehensive Stroke Rehabilitation and Research, Manipal Academy of Higher Education, Manipal, Karnataka India; 10 Sackler Faculty of Medicine, Tel Aviv University, Tel Aviv, Israel

**Keywords:** Neurological disorders, Neuroscience, Neurology

## Abstract

Hemiparesis and spasticity are common co-occurring manifestations of hemispheric stroke. The relationship between impaired precision and force in voluntary movement (hemiparesis) and the increment in muscle tone that stems from dysregulated activity of the stretch reflex (spasticity) is far from clear. Here we aimed to elucidate whether variation in lesion topography affects hemiparesis and spasticity in a similar or dis-similar manner. Voxel-based lesion-symptom mapping (VLSM) was used to assess the impact of lesion topography on (a) upper limb paresis, as reflected by the *Fugl-Meyer Assessment* scale for the upper limb and (b) elbow flexor spasticity, as reflected by the *Tonic Stretch Reflex Threshold*, in 41 patients with first-ever stroke. Hemiparesis and spasticity were affected by damage to peri-Sylvian cortical and subcortical regions and the putamen. Hemiparesis (but not spasticity) was affected by damage to the corticospinal tract at corona-radiata and capsular levels, and by damage to white-matter association tracts and additional regions in the temporal cortex and pallidum. VLSM conjunction analysis showed only a minor overlap of brain voxels where the existence of damage affected both hemiparesis and spasticity, suggesting that control of voluntary movement and regulation of muscle tone at rest involve largely separate parts of the motor network.

## Introduction

Stroke is a leading cause of adult long-term motor disability^1^, where the key symptomatology consists of contralesional hemiparesis and spasticity. More than 80% of hospitalized subjects with stroke present some degree of hemiparesis, with upper limb (UL) involvement seen in about 75%^2^. Spasticity accompanies paresis at different rates (4-43%) in different time periods post stroke^3,4^. Hemiparesis and spasticity may lead to dependence in activities of daily living and reduced quality of life^5,6^.

By ‘hemiparesis’ we refer in this article to pathological active movement comprised of reduced muscle strength and impaired movement control (precision and speed of voluntary movement) in the side of the body contralateral to the side of the stroke. The term ‘spasticity’ is used here as defined by James Lance, to denote velocity-dependent increment in muscle resistance to passive stretching, emerging from hyperexcitability of the stretch reflex arc^7^.

We have specified in this way the usage of the terms ‘hemiparesis’ and ‘spasticity’ in order to avoid confusion, as both terms are used in the clinical neurology and neuroscience literature in various ways, especially in discussions of components of the ‘pyramidal’ or ‘upper motor neuron’ syndrome and their underlying pathophysiology in humans and animals^8,9^. A word on other forms of muscle hypertonia, different from spasticity, is in place. The term ‘spastic dystonia’ was coined by Derek Denny-Brown^10^ to denote a consequence of experimental brain damage in monkeys, where muscles are activated in a sustained manner affecting the animal’s posture in a typical way. Denny-Brown reported that this phenomenon is not abolished by dorsal rhizotomy (section of neural transmission from muscle spindles which acts on spinal motor neurons and interneurons). Thus, contrary to ‘spasticity’ as defined by Lance, ‘spastic dystonia’ cannot be attributed to the effect of increased afferent stimulation of spinal motor neurons by hyperactive muscle-spindles. It should rather be conceived as a sequence of damage to motor control mechanisms within the central nervous system (an efferent phenomenon), impairing the regulation of spinal motor neurons’ excitability^10^. In a recent review by Lorentzen et al.^11^, the term ‘spastic dystonia’ was used in a much similar way, to denote ‘stretch- and effort-unrelated sustained involuntary muscle activity following central motor lesions’, which contributes to the typical hemiparetic posture. In the resting hypertonic muscle, sustained involuntary neural activation (‘spastic dystonia’) is distinguished from hypertonia due to altered mechanical properties of muscles (‘intrinsic’, ‘non-reflex’ hypertonia), by demonstration of sustained EMG activity despite intended relaxation, in the former but not in the latter state^11,12^. The important contribution of reduced mobility and disuse to the formation of intrinsic hypertonia was stressed by Dietz et al.^13^ and Gracies^14^, who proposed that the secondary soft tissue changes may affect in turn the expression of spasticity, spastic dystonia and other forms of muscle overactivity, by interaction with plastic changes occurring within the central nervous system (^15^; for a review of mechanisms underlying the different types of altered muscle tone, including spasticity and spastic dystonia, see Trompetto et al.^16^). In recent years, important information has been gathered on the relative prevalence of spasticity vs. spastic dystonia among brain-damaged patients. Marinelli and colleagues^17^ established, using surface EMG recording, the existence of spastic dystonia in the rectus femoris muscle of 8 out of 30 patients with multiple sclerosis and velocity-dependent hypertonia. The other 22 patients exhibited only spasticity, as evidenced by their ability to relax their knee extensor muscles (and abolish the EMG activity) upon request^17^. Trompetto and colleagues^18^ found a higher prevalence of spastic dystonia in the flexor carpi radialis muscle of stroke patients exhibiting velocity dependent hypertonia (in a cohort of 23 such patients, 17 had spastic dystonia and 6 had spasticity only, i.e., were able to relax the muscle at will). Recently, Puce and colleagues^19^ studied the electromyographic manifestations of hypertonic muscles in multiple sclerosis. EMG was recorded from muscles at rest, during stretch (dynamic stretch reflex) and during one minute immediately following muscle stretch (static stretch reflex). The expression of EMG activity in the three conditions differed in extensor and flexor muscles of the upper and lower limbs, providing novel insight into the heterogeneity of muscle hypertonia states in the upper motor neuron syndrome^19^.

The precise relationship between spasticity and hemiparesis following stroke is far from clear. In motor-control theorizing, the *Threshold Control* (or *Referent Control*) *Theory* suggests that the nervous system controls posture and movement by specifying, in a task sensitive manner, a spatial point in the biomechanical range of a joint (muscle length/joint angle) where the stretch-reflex is activated and motoneuronal recruitment begins^20–24^. By stretching a completely relaxed muscle at different velocities, and observing the dynamic spatial threshold for the emergence of reactive contraction at each velocity, one can extrapolate the *Tonic Stretch Reflex Threshold* (TSRT), denoting the angular threshold for muscle spindle reflexive activation and the emergence of reactive muscle contraction in the resting state. To maintain a muscle relaxed throughout the entire biomechanical range, the TSRT has to be shifted outside of the biomechanical range^25^. Following brain damage, the ability to regulate the spatial thresholds for muscle contraction in a task-specific manner is impaired. In patients with spastic hemiparesis this impairment may lead to the inability to shift the TSRT outside of the biomechanical range in order to voluntarily relax muscles^26^. Consequently, attempts to passively or actively move the limb beyond the pathologically set TSRT evoke increased resistance, or spasticity. The *Threshold Control Theory* predicts that impaired regulation of motoneuronal excitation thresholds will lead both to spasticity and arm paresis^26–30^. The neural substrate involved in task-dependent threshold setting is largely unknown^21^.

From a clinical perspective on the relationship between hemiparesis and spasticity, patients with elbow/wrist spasticity, measured with the Modified Ashworth Scale (MAS), were found to have significantly lower scores on the Fugl-Meyer Assessment scale of the hemiparetic UL (FMA-UL) compared to patients without elbow/wrist spasticity^31^. The MAS subjectively grades the amount of resistance felt during passive stretching of muscles on a 6-point ordinal scale^32,33^ and the FMA-UL tests the patient’s ability to execute different movements in a precise manner^34^. The MAS was found also to relate to muscle strength, such that patients with less strength in proximal and distal muscles of the hemiparetic UL (measured by the British Medical Research Council scale) had a higher risk of developing spasticity as reflected by the MAS^35^, and poor strength in the hemiparetic UL (measured with Upper Extremity Motricity Index) on admission to rehabilitation was the most effective predictor of moderate to severe spasticity measured by the MAS^36^. Recently, our group showed links between the severity of spasticity post stroke (as reflected by the TSRT and the MAS) and stochastic measures of the distance between pathological and normal elbow movement. Thus, subacute stroke patients with spasticity exhibited reaching motion that was less similar to the motion of healthy subjects. This was reflected by high bi-directional Kullback-Liebler divergence and Hellinger’s distance measures^37,38^.

On the other hand, there is empirical evidence which points to non-correlated and even discordant relations between paresis and spasticity post stroke. One often sees patients with complete hemiplegia who are hypotonic in the paretic side. Spasticity develops only in about a third of hemiparetic patients and severe paresis is seen almost as often in patients with and without spasticity^39^. The idea that spasticity contributes to the development of hemiparesis is based largely on the hypothetical assumption that the attempted voluntary activation of the agonist muscle in a joint causes the hyper-excitable reflex arc in the antagonist muscle to react by co-contraction, thus slowing down or even preventing the intended movement (‘co-contraction paresis’). However, slow and abnormal elbow movements made by patients with chronic stroke were shown to be more related to the degree of maximal isometric torque that the agonist muscle could generate than to the level of muscle spasticity in the antagonist. Actually, antagonist activity during movement was at or below the normal range even in patients with marked hypertonus at rest in the antagonist muscle^40^. Spasticity was found to explain only part of the deviation from normality observed in movement kinematics. Thus, peak reaching velocity in the hemiparetic UL was shown to be affected mainly by the reduction in muscle strength, rather than by the level of spasticity (averaged MAS values from muscles acting at the shoulder, elbow and wrist joints). End-point error and reach path curvature were influenced mainly by individuation index, with spasticity explaining a smaller portion of the variance^41^.

The impact of variation in stroke lesion topography on the expression of hemiparesis was studied extensively^42–48^. Some studies addressed also lesion effects on spasticity^49–52^. Hemiparesis was associated in most cases with damage to white-matter regions containing the corticospinal tract (CST; corona radiata and posterior limb of internal capsule) and association fibers (e.g., superior longitudinal fasciculus), sensory-motor cortex, insular and peri-Sylvian opercular cortical regions and the putamen^42–48^. Spasticity was found to relate to damage to the insula, premotor cortex, thalamus, basal ganglia, and white matter regions including the internal capsule, corona radiata and external capsule^49–52^. However, these studies did not disclose the extent of network sharing between hemiparesis and spasticity at the voxel level. As each of the above structures is comprised of hundreds and thousands of brain voxels, hemiparesis and spasticity could be affected by same or different voxels within a given structure.

Past studies of lesion effects on spasticity used the MAS^32,33^ as a marker of muscle hypertonia. The MAS, which is widely used as a clinical measure of spasticity, is based on subjective assessment by the examiner of the amount of resistance felt when the limb is passively stretched. Passive stretching is done usually at a non-specified velocity, despite the strong velocity dependence of hyper excitable stretch reflex activity assumed to underly spasticity^7,53^. The validity of the MAS has been questioned^33,54–57^ and the reported values for MAS inter-rater and intra-rater reliability are variable (inter-class correlation coefficients [ICCs range = 0.54–0.85]^32,33,54,58–60^.

In the current study we aimed to determine the impact of variation in lesion topography on paresis and spasticity of the contralesional UL. Using voxel-based lesion symptom mapping (VLSM^61^), we identified brain voxels where the existence of damage affected the severity of hemiparesis (as reflected in the FMA-UL score) and brain voxels where the existence of damage affected the severity of spasticity (as reflected in TSRT measurement). This is the first time TSRT rather than the MAS is used in a lesion study of spasticity. VLSM conjunction analysis was applied to assess the extent of network sharing at the voxel level. As the study was conducted in the subacute period following stroke, we hypothesized that both paresis and spasticity would be affected by damage to brain structures involved in the control of movement in the intact brain, as well as structures supporting motor recovery processes. Given the evidence for often discordant relations between the magnitude of hemiparesis and spasticity^8,31,35,36,39–41^, we hypothesized that VLSM conjunction analysis may reveal both common and distinct brain voxels affecting these two components of the upper motor neuron syndrome. 

## Methods

### Participants

Forty-one first-event stroke patients who sustained a stroke 2.2 ± 1.3 months previously participated. Data were collected in the framework of the ENHANCE study^62^. Patients were included if they (1) had a single, first-event stroke in middle cerebral artery territory, confirmed by magnetic resonance imaging or computed tomography; (2) were in a stable clinical and metabolic state; (3) were 25–80 years of age; (4) were in the sub-acute phase of the disease (3 weeks to 6 months post stroke onset); (5) had arm paresis (2–6/7 on the Chedoke-McMaster Stroke Assessment arm score^63^), but were able to perform at least 30° voluntary elbow flexion and extension; (6) had elbow flexor spasticity; (7) were able to provide informed consent. In order to minimize variance in premorbid structure–function relationship we aimed to recruit only right-handed persons, but eventually only 37 of the 41 participants comprising the study cohort were right handers and four were actually ambidextrous. Exclusion criteria were the presence of other neurological or major neuromuscular/orthopedic problems, pain, or difficulty comprehending instructions. All experimental protocols were approved by the Institutional Review Board/Ethics Committee of Loewenstein Rehabilitation Medical Center, Raanana, Israel; Institutional Ethic Committee of the Tel Aviv University, Tel-Aviv, Israel; Center for Interdisciplinary Research in Rehabilitation, Montreal, Canada; Institutional Ethics Committee of Kasturba Hospital, Manipal, India; and Manipal University, Manipal, India. All experiments were performed in accordance with relevant guidelines and regulations. Participants signed informed consent forms approved by institutional review boards of Loewenstein Rehabilitation Medical Center, Raanana, Israel; Institutional Ethic Committee of the Tel Aviv University, Tel-Aviv, Israel; Center for Interdisciplinary Research in Rehabilitation, Montreal, Canada; Institutional Ethics Committee of Kasturba Hospital, Manipal, India; and Manipal University, Manipal, India. The current cohort included stroke patients who participated also in our recently published study^64^, but only those who met the aforementioned inclusion criteria. Individual patients’ data are shown in supplementary Table S1.

### Hemiparesis assessment (FMA-UL)

The standardized sensitive, reliable and valid FMA-UL^34,65,66^ was used for evaluation of hemiparesis. The FMA-UL contains 33 test items for the hemiparetic UL. These items are divided into four subsections: shoulder-arm, wrist, hand, and upper-limb coordination. Each test item is scored on a 3-point ordinal scale (0 = no movement, 1 = partial movement, 2 = full movement), with a maximal total score of 66 points.

### Spasticity assessment (TSRT and MAS)

The Montreal Spasticity Measure (MSM; for details see^64^) determined the TSRT in the Biceps Brachii (BB) muscle from a series of dynamic stretch reflex responses evoked by a clinician stretching the elbow flexors at different velocities. After preparing the skin, active bipolar electrodes (Procomp 5; Thought Technology, Montreal, QC, Canada) were placed above the bulk of the BB (outer, long head) and the Triceps Brachii (lateral head) based on known anatomical landmarks and palpation. A high-resolver electrogoniometer (servo-type rotational-position potentiometer P2200; Novotechnik U.S. Inc., Southborough, MA, USA) was attached with straps to the lateral aspect of the arm and forearm with the axis of rotation aligned with the lateral epicondyle. Participants sat with the arm resting in slight abduction on a pillow. At the start of testing, participants performed an isometric maximal voluntary contraction of elbow flexors to adjust the electromyography (EMG) gain. The elbow starting angle was set to the maximal flexion allowed by the approximation of the arm and the forearm but without contact between the two segments (± 10°). The forearm was in the neutral position between pronation and supination. The baseline EMG signal and the initial elbow angle were reproduced prior to each stretch. A trial consisted of stretching the forearm from the initial position towards full extension (180°) and returning the forearm to the starting position. Before each stretch, a randomly assigned speed, equally distributed between slow, moderate and fast velocities, was indicated to the evaluator by an auditory signal from the MSM. Stretch velocities were randomized to avoid anticipation of the upcoming stretch velocity. Twenty stretches were performed with a resting period of at least 10 s between stretches to minimize effects of stretch-reflex habituation^67,68^. Participants were instructed to relax completely. For each stretch, the MSM identifies the angle and velocity at which the evoked stretch response is elicited (this is the point where the EMG signal of the stretched muscle increases and remains 3SDs above the background EMG level for 25 ms). The TSRT angle (at rest) is calculated by extrapolation of a linear regression line through these dynamic angle/velocity thresholds to zero velocity. More severe spasticity is expressed by a smaller TSRT angle. A detailed description of TSRT recording and analysis is provided in Frenkel-Toledo et al.^64^.

Spasticity of the BB was also assessed by the MAS, as a background measure, as past lesion studies of spasticity used this test (see Introduction). The MAS is measured on a six-point ordinal scale (0, 1, 1+ , 2, 3, 4) to subjectively rate the resistance to passive stretching. In the current study we aimed to maintain a stretching velocity of approximately 100°/s^32^.

### Lesion analysis

Delineation of lesion boundaries was done manually on digitized follow-up brain CT or MRI scans, dated on average 30 days post stroke onset, by a physician highly experienced in analysis of neuro-imaging data, who was blinded to all other participants' information (author NS). Lesion analyses were performed with the Analysis of Brain Lesions (ABLe) module implemented in MEDx software (Medical-Numerics, Sterling, VA, USA). ABLe characterizes brain lesions in MRI and CT scans of the adult human brain by spatially normalizing the lesioned brain into Talairach space using the Montreal Neurological Institute (MNI) template. It reports tissue damage in the normalized brain using an interface to the Talairach Daemon (San Antonio, Texas)^69^, Automated Anatomical Labeling (AAL) atlas^70,71^, Volume Occupancy Talairach Labels (VOTL) atlas^69,71^ or the White Matter Atlas^72^. Quantification of the amount of tissue damage within each structure/region of the atlas was obtained as described earlier^73^. In the current study, tissue damage in the normalized brain was reported using the interface to the AAL and white matter atlases. Registration accuracy of the scans to the MNI template^71^ across all subjects ranged from 89.2% to 94.2%.

### Voxel-based lesion-symptom mapping (VLSM)

VLSM^61^ was used to identify voxels (2 × 2 × 2 mm) of the normalized brain where the existence of damage has a significant impact on the FMA-UL as a measure of UL paresis, and the BB TSRT as a measure of elbow flexor spasticity. Voxel-by-voxel analysis was used to calculate the statistical significance of performance difference between subjects with and without damage in a given voxel, using the Mann–Whitney test (due to non-normal group distribution of data). Only voxels damaged in at least 20% of the subjects were tested, and at least 10 adjacent voxels had to show a statistically significant impact of damage on performance for a cluster of voxels to be reported^43,74–76^. To correct for multiple comparisons, voxels with values exceeding a false discovery rate (FDR) of p < 0.05 were considered significant^76,77^. However, due to insufficient statistical power in the VLSM analysis of TSRT, the results in this analysis did not survive the FDR correction for multiple comparisons and we report for this analysis anatomical regions containing clusters of at least 10 voxels, where patients affected in these voxels showed disadvantage relative to patients who were not affected in these voxels, using a lenient criterion of p < 0.018 (corresponding to z score equal to or higher than 2.09, which is the minimal z score of voxels that passed the FDR correction for multiple comparisons in the FMA-UL analysis. For a similar approach see^33,35,74,78–80^). The maximum z-score is reported for each cluster of contiguous above-threshold voxels. Since there may be multiple voxels with this maximum z-score in the cluster, we report the coordinates of the voxel that is most superior, posterior and left in its location within the cluster (the centroid of the cluster is not reported as it may not have the highest z-score value and it may not be an above-threshold voxel). The AAL atlas for gray matter and the White Matter Atlas^69–72^ were used to identify the brain structures in which the significant clusters were located. VLSM conjunction analysis was used to classify ‘significant’ voxels (voxels with z scores equal to or higher than 2.09) as affecting (1) both the FMA-UL and TSRT; (2) only the FMA-UL scores; and (3) only the TSRT scores. Because of the relatively small sample size of the cohort, VLSM and conjunction analyses were conducted on the entire cohort, after flipping the scans onto a single (left) hemisphere template. The results after flipping onto the right hemisphere are very similar except for some differences in the number of voxels in some regions due to subtle differences in the extent of homologous regions in the two hemispheres.

## Results

Demographic and clinical information as well as FMA-UL and TSRT scores are described in Table 1. Elbow flexor TSRT scores did not correlate with the FMA-UL scores (Spearman’s rho = −0.066, p = 0.683) nor with MAS scores (Spearman’s rho = 0.130, p = 0.420) and the FMA-UL scores did not correlate with the MAS scores (Spearman’s rho = −0.116, p = 0.471).

**Table 1:** Demographic and clinical characteristics of participants.

	#/#, mean ± SD (range)
Sex (male/female)	25/16
Age (years)	55.2 ± 10.8 (38–77)
Motor dominance (R/A)	37/4
Lesioned hemisphere (R/L)	20/21
Lesion type (I/H/I > H)	31/9/1
TAO (months)	2.2 ± 1.3 (0.6–5.9)
Lesion volume (cc)	31.7 ± 44.7 (0.4–182.3)
FMA-UL	32.3 ± 12.2 (14–54)
TSRT (°)	107.4 ± 25.7 (61.7–162.7)

*SD* standard deviation, motor dominance *R* right, *A* ambidextrous, *FMA-UL* Fugl-Meyer assessment scale for the upper limb (maximal score—66); *lesion type I* ischemic, *H* hemorrhagic, *I > H* ischemic with hemorrhagic transformation, *TAO* time after stroke onset, *TSRT* tonic stretch reflex threshold.

**Figure 1:**
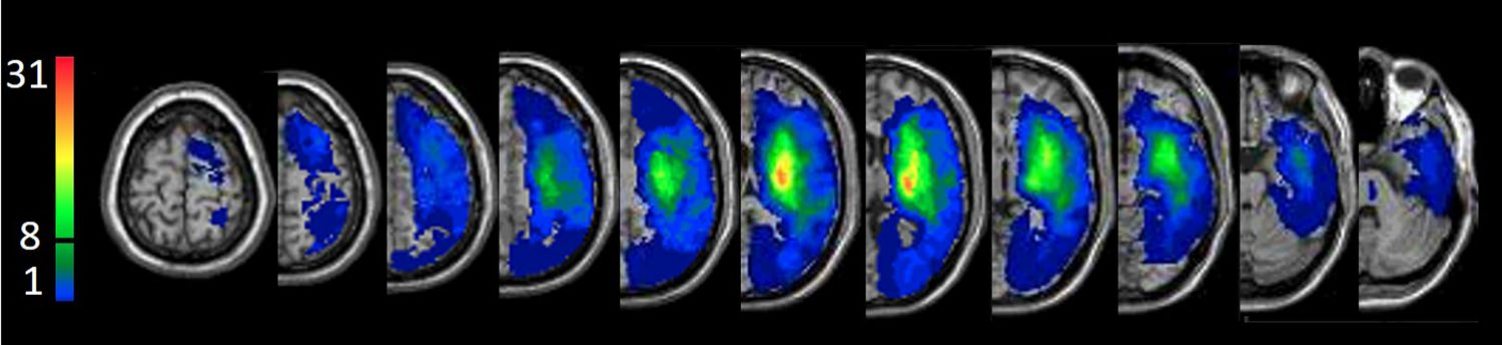
Lesion overlay map of the entire cohort (n = 41) after flipping right-hemisphere lesions to the left. At least 8 subjects (20%) had to have damage to a particular voxel for it to be included in the VLSM analysis. Representative normalized slices (out of 90 normalized slices employed) are displayed in radiological convention (left hemisphere on right), with warmer colors indicating greater lesion overlap (units: number of patients with lesion in this region).

Overlay lesion map (stroke lesion distribution) of the entire cohort is shown in Fig. 1.

**Figure 2:**
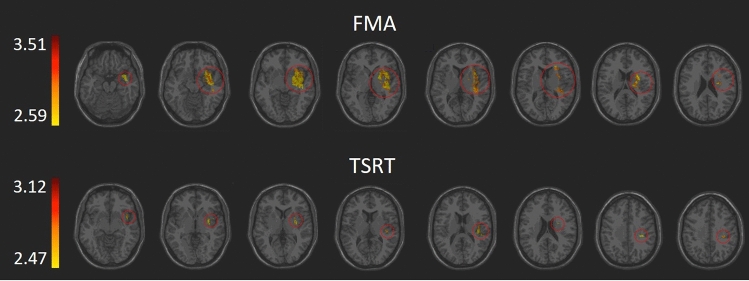
VLSM analysis of the entire cohort (n = 41; right hemisphere lesions flipped to left hemisphere) depicting areas where the existence of damage was associated with significantly lower FMA/TSRT scores (minimum cluster size—10 contiguous voxels, minimum number of patients affected in a voxel—8). Warmer colors indicate higher z-scores. The colored regions in the FMA analysis survived FDR correction for multiple comparisons, and the colored regions in the TSRT did not, but represent a lenient criterion of significance where z score ≥ 2.09 (p ≤ 0.018), similar to the minimal z score of voxels surviving the FDR correction for multiple comparisons in the FMA analysis.

VLSM analysis^53^ identified clusters of voxels where the existence of damage was associated with significantly more severe UL paresis (lower FMA-UL scores) and greater BB spasticity (lower TSRT values) in the contralesional UL (Fig. 2). The anatomical structures containing the ‘significant’ voxels are shown in Table 2. The overall number of ‘significant’ voxels (2161 and 265 for FMA-UL and TSRT, respectively) and the number of these voxels among different cortical and subcortical structures, were much higher in the FMA-UL analysis compared to the TSRT analysis. Even when a structure contained ‘significant’ voxels in both FMA-UL and TSRT analyses (insular cortex, putamen, superior temporal gyrus, Heschl gyrus, Rolandic operculum and external capsule), the number of voxels where the existence of damage affected significantly the FMA-UL scores largely outnumbered the number of voxels in which damage affected the TSRT scores. All the structures that contained brain voxels ‘significant’ for TSRT scores emerged also in the FMA-UL VLSM analysis. However, patients’ FMA-UL scores were additionally affected in a selective manner by damage to the CST (in its passage through the corona radiata and the posterior limb of the internal capsule), as well as damage to white matter association tracts (inferior fronto-occipital, superior longitudinal and uncinate fasciculi), regions in the temporal cortex and pallidum.

**Table 2:** VLSM analysis for FMA-UL and TSRT in the entire cohort (n = 41).

Test	Structure	Z-value	X	Y	Z	Voxels	% area
FMA-UL	Insula	3.51	−28	10	−14	847	45.59
	Putamen	3.38	−24	2	−6	302	29.93
	STG	3.37	−42	4	−10	244	10.63
	EC	3.38	−30	12	−2	172	38.22
	IFO	3.47	−26	10	−12	168	69.42
	Heschl gyrus	3.04	−44	−12	4	80	35.56
	Pallidum	3.34	−20	4	−6	72	24.57
	SCR	2.90	−30	−8	22	50	5.41
	PLIC	3.27	−26	−8	18	37	7.76
	ACR	3.04	−24	30	14	32	3.70
	SLF	3.16	−32	2	18	31	3.80
	SS	3.21	−36	−14	−10	25	8.68
	R. operculum	2.59	−36	6	14	25	2.53
	UNC	3.07	−34	4	−16	23	46.94
	RLIC	3.14	−30	−28	10	20	6.43
	Temporal Pole Sup	3.39	−40	6	−18	19	1.48
	MTG	2.90	−48	−12	−12	14	0.28
TSRT	R. operculum	2.93	−38	−28	14	78	7.88
	EC	3.12	−32	−8	6	56	12.44
	Putamen	3.10	−32	−2	−2	47	4.66
	STG	2.57	−44	−22	8	39	1.70
	Insula	2.83	−44	6	−6	17	0.91
		2.47	−40	−10	−12	16	0.86
	Heschl gyrus	2.47	−38	−26	14	12	5.33

VLSM of FMA-UL passed FDR correction for multiple comparisons (corresponding in these analyses to z scores equal to or higher than 2.09). VLSM results of TSRT did not survive the FDR correction and are based on a lenient criterion (z score = 2.09 or above corresponding to uncorrected p ≤ 0.018).

*FMA-UL* Fugl-Meyer assessment scale for the upper limb, *TSRT* tonic stretch reflex threshold, *ACR/SCR* anterior/superior corona radiata, *EC* external capsule, *IFO* inferior fronto-occipital fasciculus, *MTG* middle temporal gyrus, *PLIC/RLIC* posterior/retro-lenticular limb of internal capsule, *R. operculum* Rolandic operculum, *SLF* superior longitudinal fasciculus, *SS* sagittal stratum, *STG* superior temporal gyrus, *Temporal Pole Sup* temporopolar region of STG, *UNC* uncinate fasciculus.

**Figure 3:**
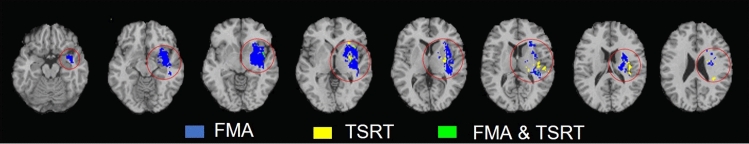
VLSM conjunction analysis in the entire cohort (n = 41), after flipping right hemisphere lesions to the left. Colored regions depict areas of brain damage that were associated with significantly lower scores in the FMA only (blue), TSRT only (yellow), and both FMA and TSRT (green).

Table 3 and Fig. 3 show the anatomical structures where VLSM conjunction analysis disclosed brain voxels (not necessarily in clusters of ≥ 10 voxels, as in Table 2) in which the existence of damage affected significantly only the FMA-UL scores, only the TSRT scores, and both the FMA-UL and TSRT scores. In the large majority (2078 of 2162 voxels; 96%) of FMA-UL “significant” voxels, the effect on hemiparesis was selective and damage to these voxels did not affect the magnitude of spasticity, as reflected in BB TSRT values. In the majority (248 of 332 voxels; 75%) of TSRT “significant” voxels the impact of damage on BB spasticity was selective and damage to these voxels had no effect on FMA-UL scores. Thus, the VLSM conjunction analysis shows only a minor level of anatomical overlap between brain voxels related to paresis and brain voxels related to spasticity. VLSM conducted on spasticity measured by the MAS failed to disclose voxel clusters where the existence of damage affected the test scores in a significant manner.

**Table 3:** VLSM conjunction analysis for FMA-UL and TSRT in the entire cohort (n = 41).

Areas	FMA-UL only	TSRT only	FMA-UL plus TSRT
Insula	817	27	30
Putamen	284	30	18
STG	234	38	10
EC	146	35	26
Heschl gyrus	80	12	
R. operculum	25	76	
SCR	51	18^a^	
SLF	32	12^a^	
IFO	168		
Pallidum	72		
PLIC	43		
ACR	35		
SS	25		
UNC	23		
RLIC	20		
Temporal Pole Sup	19		
MTG	14		

Number of voxels in affected brain regions where damage had a significant impact on FMA-UL only (z = 2.09 or higher), TSRT only (z = 2.09 or higher), and FMA-UL plus TSRT, in the entire cohort (n = 41). Only structures with at least 10 voxels affecting performance in one or more of the 3 options are shown.

*FMA-UL* Fugl-Meyer assessment scale for the upper limb, *TSRT* tonic stretch reflex threshold, *ACR/SCR* anterior/superior corona radiata, *EC* external capsule, *IFO* inferior fronto-occipital fasciculus, *MTG* middle temporal gyrus, *PLIC/RLIC* posterior/retro-lenticular limb of internal capsule, *R. operculum* Rolandic operculum, *SLF* superior longitudinal fasciculus, *SS* sagittal stratum, *STG* superior temporal gyrus, *Temporal Pole Sup* temporopolar region of STG, *UNC* uncinate fasciculus.

^a^These structures did not appear in the VLSM analysis of the TSRT in Table 2 as at least 10 adjacent voxels had to show a statistically significant impact on performance for a cluster of voxels to be reported^33,65,66^.

## Discussion

The aim of the current study was to comparatively assess the impact of lesion topography on two components of the upper motor neuron syndrome—paresis and spasticity. We used VLSM to determine the pattern of brain damage connected with UL paresis (FMA-UL) vs. spasticity (TSRT of the BB). To our knowledge, this is the first attempt to investigate the effects of lesion topography on hemiparesis vs. spasticity at the voxel level. This is also the first analysis of the impact of variation in lesion topography on the TSRT.

The number of brain voxels in which the existence of damage had an impact on the severity of UL paresis (FMA-UL score) was much larger than the number of brain voxels in which the existence of damage had an impact on spasticity (BB TSRT). Moreover, the number of cortical regions and subcortical structures hosting the ‘significant’ voxels, was much larger in the case of hemiparesis compared to spasticity (Table 2).

At the time of testing (subacute phase, first months post stroke onset), UL paresis was related to damage in brain voxels residing in centrally located cortical regions (insula, superior and middle temporal gyri, Rolandic operculum), the CST (in its passage through the corona radiata and the posterior limb of the internal capsule), different white-matter association tracts (inferior fronto-occipital, superior longitudinal and uncinate fasciculi and the sagittal stratum), and the putamen and pallidum parts of the basal ganglia. These results are in line with earlier lesion studies of hemiparesis^42,47,48^ and our own study in a different cohort^43^. The findings point to the central role of the ipsilesional CST in mediation of cerebral control over the spinal motor activity that drives movement in the hemiparetic UL^8,81^. The evidence for that from lesion studies is corroborated by motor evoked potentials research^82,83^. The significant impact of damage to centrally located cortical regions outside the primary motor cortex (M1) and the salient impact of damage to white matter association tracts and the basal ganglia, point to the wide network that supports the planning and execution of voluntary movement^84–86^ and to the involvement of various network components in the re-organization of cerebral motor control occurring in the subacute period (first months) following a stroke^87,88^. Plastic changes taking place at this time should relate to various components of motor activity, such as integration of somato-sensory and visuospatial information into motor plans, and acquisition of new motor skills through procedural learning. This suggests that brain regions beyond the premotor and motor cortices take part in the process^89^. Notable among the cortical components of the re-organizing network is the insula, in which the existence of damage to almost half of its voxels was found to affect patients’ FMA-UL scores. This finding points to an important role of the insular cortex in motor behavior^90,91^. The insula is known to be involved in the execution of arm movements in the gravitational field^92^. The insular cortex and temporal pole were shown to encode movement direction in a center-out motor task^93^, and the middle temporal gyrus was shown to be involved in encoding path-related information during a center-out motor task^94^. The involvement of nonmotor regions such as insula, temporal pole and the middle and superior temporal gyri point to their role in adaptive remapping processes supporting recovery^95^. Our finding pointing to the importance of the superior longitudinal fasciculus, is in line with past demonstration that microstructure of this major white-matter association tract predicts stimulation-induced interference with on-line motor control^96^. Also note that damage to M1 voxels was not shown in the current VLSM analysis to have a significant impact on hemiparetic UL movement. This somewhat unexpected finding is explained by the relatively infrequent involvement of the more dorsal parts of the precentral gyrus, hosting the M1 hand and arm areas, in the stroke process, as can be seen in the lesion overlay map of the current cohort (Fig. 1). With involvement of at least 10–20% of the cohort, usually set as threshold for voxel inclusion in the VLSM analysis^43,75,76^, damage to dorsal precentral gyrus voxels is often under-represented, a fact leading to false negative results with respect to this region.

Spasticity, as reflected in BB TSRT was affected by damage to a much smaller number of brain voxels, residing in a smaller array of cortical and subcortical structures, compared to UL hemiparesis. In the case of spasticity, VLSM disclosed ‘significant’ voxels in peri-Sylvian cortical regions (the Rolandic operculum, superior temporal gyrus and the insula) and in the external capsule and putamen. Thus, the anatomical network related to spasticity was more restricted than the network related to hemiparesis, despite the use of a more lenient criterion of significance in the case of spasticity, as the results of the VLSM analysis for spasticity did not survive the FDR correction for multiple comparisons. In previous research, spasticity was also found to relate to damage in the insular cortex, basal ganglia and external capsule^49–52^. The putamen was identified as the site most tightly associated with spasticity in a VLSM study by Cheung and colleagues^50^. Yet, in contrast to the findings of the current research, in these earlier studies, spasticity was related also to damage in the premotor cortex, thalamus and white-matter projection (corona radiata and posterior limb of the internal capsule) and association (superior longitudinal fasciculus) tracts—a distribution of areas quite similar to our findings with respect to UL paresis. These studies used the MAS as a measure of spasticity, whereas we used TSRT. Moreover, in the earlier studies, spasticity was assessed in different proximal and distal UL muscles, whereas in the current study, only spasticity generated in the BB was used. These differences may explain the more restricted network emerging in the current study, relative to the previous studies^49–52^. As mentioned before, TSRT and MAS assess different aspects of spasticity. TSRT is a measure derived from a specific line of motor control theorizing (*Threshold or Referent Control Theory*^21,22,26^), where the severity of impairment in regulation of stretch reflex activity is represented by the spatial threshold (angle) in the joint range of motion at which the hyperactive stretch reflex begins to act. The MAS, in contrast, measures the amount of supra-threshold resistance produced by the stretched muscle^53^, without considering the joint angle at which pathological resistance begins. In past research, TSRT values did not correlate with MAS scores in spastic elbow flexor muscles, in patients assessed at the sub-acute^64^ and chronic^97^ stages post stroke onset. In the current study, VLSM of MAS results did not yield voxel clusters where the existence of damage exerted a statistically significant effect on the test scores (even when employing a lenient criterion of significance, as done here for the TSRT). This divergence from past lesion studies using the MAS is probably related to the fact that patients had to be capable of producing 30° flexion–extension elbow movements with the hemiparetic UL in order to be recruited to the current study. Consequently, the cohort here did not include patients with severe spasticity in elbow flexors (MAS scores ranged between 1 and 2 in all patients; Table S1).

As the assessment of muscle hypertonia in the current study is based on MAS and TSRT measurements only, i.e., measurements informative on properties of the dynamic component of the tonic stretch reflex (how the muscle behaves during the time period of the stretch), it is impossible to exclude the possibility that in part of the cohort muscle hypertonia was contributed by spastic dystonia. Note that the measurement of the TSRT was based on determination of the point where the EMG signal of the stretched muscle increased and remained 3SDs above the background EMG level for 25 ms. The patient was required to relax the elbow as much as possible prior to the stretch, in order to permit a reliable assessment of the spatial location where this EMG dynamics occurred during the stretch. Despite the fact that MAS scores in the cohort did not surpass the lower range (1–2), in part of the patients the background EMG was not abolished completely during the attempted relaxation prior to the stretch. Thus, although we did not employ a standardized recording of surface EMG at rest, as done in order to establish the presence of spastic dystonia^17,18^, we should assume that in part of the patients, velocity-dependent increment in muscle resistance to passive stretching, emerging from hyperexcitability of the stretch reflex arc (i.e., spasticity^7^), was accompanied by co-occurring involuntary tonic activation of resting muscles (spastic dystonia^11,16^). The anatomical network identified here by VLSM of TSRT results may have been influenced by the co-occurrence of spastic dystonia. In stroke patients in whom neurally-mediated muscle hypertonia of the UL results from spasticity only—a minority of the stroke population according to Trompetto et al.^18^—the anatomical network is likely to be more restricted.

The results of the VLSM conjunction analysis, stratifying brain voxels where damage affects hemiparesis only, spasticity only, or both hemiparesis and spasticity (Fig. 3, Table 3), shed further light on the neuroanatomical substrate underlying these two components of the upper motor neuron syndrome. The voxels in which the existence of damage had an effect on both paresis and spasticity comprise about 25% and 4% of the total number of voxels affecting spasticity and arm paresis, respectively. Thus, the analysis of lesion-behavior relationship, conducted here at the voxel level, shows that even in cases where the same anatomical structure is implicated in both behaviors, the majority of ‘significant’ voxels had a selective impact on either arm paresis or spasticity but not on both. The number of voxels where damage affects arm paresis is much larger than the number of voxels where damage affects the magnitude of spasticity (with the exception of Rolandic operculum showing the opposite trend).

The presence, within the trajectory of the CST, of brain voxels in which the existence of damage affects the severity of hemiparesis, is expected, given the crucial role played by the CST in mediation of cerebral control over voluntary movement^8,98,99^. On the other hand, the absence there of voxels in which the existence of damage affects the severity of spasticity (in the sense of deviation from normality in setting the spatial threshold for the recruitment of muscle-spindle mediated reflexive contraction at stretching), raises questions on the role of CST damage in spasticity.

The underlying mechanism of spasticity and its relation to other components of the ‘upper motor neuron’ or ‘pyramidal’ syndrome is a matter of long-lasting debate^8,9^. One key question is whether CST damage, which is widely recognized as the main determinant of the severity of hemiparesis^8,81^, also plays a direct role in the formation of spasticity. Demonstrations of paresis, or at least the loss of precision (dexterity) in voluntary movement—without spasticity—following experimental lesioning confined to the CS tracts in macaque monkeys^100–102^ and other animals^103^ were taken as empirical evidence that CST damage is not the prime cause of spasticity^104–106^. There are case reports pointing to the emergence of hemiparesis without spasticity following damage confined to the CST in humans^107,108^. However, most of the empirical evidence in favor of a non-pyramidal mediation of spasticity comes from animal research, and claims have been made against direct extrapolation from even high non-human primates to humans, given the significant variation in functional neuroanatomy of descending projection tracts between the species^9,98^. As said, a different aspect of the severity of spasticity, not expressed in the TSRT value, i.e., the magnitude of resistance to passive stretching, as assessed by the MAS, was shown in past lesion studies to have a more extensive anatomical correlate compared to our TSRT anatomical correlates, including an effect for pyramidal tract damage^49,51,52^.

The *Threshold Control* (or *Referent Control*) *Theory*^21,22,25,26^ which forms the theoretical foundation for using the TSRT as a measure of spasticity, offers testable predictions concerning a wide range of normal and pathological motor behaviors^21^. However, the neural mediators of task-sensitive referent control are far from clear. In the current study, the functional anatomy of referent setting was tested, for the first time in the brain-voxel resolution level, with respect to muscle tone regulation at rest. Pathological setting of the spatial threshold for muscle-spindle recruitment by passive stretching was found to relate to a number of brain structures—the Rolandic operculum, superior temporal and insular cortical regions and the external capsule and putamen. These structures contained voxel clusters where the existence of damage affected the severity of spasticity as reflected by the TSRT. Damage to a minority of these voxels also affected the severity of hemiparesis, possibly pointing to some commonality in the cerebral mechanism mediating the implementation of referent control in voluntary movement as well as in muscle tone regulation. Overall, our findings support the view that the cerebral neural network controlling voluntary movement is much larger than the cerebral network controlling muscle tone at rest. Our findings seem to show that in conditions of hemispheric stroke, damage to the pyramidal tract in itself is insufficient to evoke the aspect of spasticity reflected in the TSRT value. In terms of the *Referent Control Theory*, this would be the magnitude of deviation from normality when setting the spatial threshold for muscle-spindle signaling in the resting muscle.

Spasticity, viewed as a manifestation of hyper-excitability of the stretch reflex arc^7^, is widely thought to emerge from failed balancing of excitatory and inhibitory descending signals of supraspinal origins, though much is still uncertain in this respect^104–107,109^. One suggestion emphasizes the role of the ‘extrapyramidal’ reticulospinal and vestibulospinal descending tracts (RST, VST). Thus, the dorsal (lateral, medullary) RST is thought to provide an inhibitory effect on the stretch reflex, while the medial (pontine) RST and the VST provide excitatory inputs to the stretch reflex. The dorsal RST receives facilitatory stimulation from the contralateral motor cortex via corticoreticular projections, which run in close proximity to the CST and are often affected together by the stroke process. Reduced inhibition from the dorsal (medullary) RST and/or increased excitation from the unopposed medial (pontine) RST and VST (which lack contralateral cortical control) lead to hyperexcitability of the muscle spindle reflex arc and to muscle hypertonia^104–106^. However, in decerebrated cats in whom the VST was destroyed (in the floor of the fourth ventricle), the limbs in the side as the VST lesion were flaccid, the stretch reflex in the knee extensor muscles was diminished or absent, and the ipsilateral knee jerk was pendular, prolonged and more readily inhibited than that on the opposite side^110^. The current lesion study cannot inform much on the specific contributions of brainstem descending pathways to regulation of muscle tone at rest. As all the patients recruited for this study had hemispheric strokes in MCA territory, all posterior fossa structures, including the cell assemblies in the tegmentum of the brainstem from which descend the above tracts, were not involved in the stroke process.

Interpretation of lesion analyses concerning the neural substrate of a given behavior should take into consideration the time interval between the occurrence of structural damage (stroke onset) and behavior assessment^111^. The FMA-UL and TSRT were assessed here in the sub-acute period (2.2 ± 1.3 months after onset), when the patients were hospitalized for rehabilitation. In the subacute period, behavioral improvement is most salient, reflecting resolution of secondary damage and edema, and improved physiological state in peri-lesional cortex where adaptive remapping processes take place^112–116^. Therefore, task performance at the early sub-acute phase is influenced, on one hand, by the relation of lesion topography to the pre-morbid localization of the neural network that supports the tested behavior, and on the other hand, by the emerging effects of natural and treatment-related neuroplasticity. Lesion effects on behavior tested later, in the chronic phase, differ from the effects observed in the acute and subacute periods, because the impact of damage to the relevant pre-morbid neural network subsides with the maturation of functional re-mapping and reorganization processes which take place in peri-lesional and other brain regions^111,116^. Thus, the behavioral impact of damage to a given brain structure is more likely to reflect the natural structure–function relationship when task performance is measured shortly rather than lately after the onset of stroke^111^.

Several limitations in the study should be acknowledged. First, the current analysis precluded the assessment of lesion impact on additional, potentially relevant, brain regions, because: (a) only voxels damaged in at least 20% of the cohort entered the VLSM analysis (this threshold was set in order to lower the occurrence of false positive results). Given the number of subjects in the current cohort (n = 41), it was not possible to assess the impact of damage to relevant brain voxels of the more dorsomedial parts of the sensory-motor cortex, including the hand and arm parts of M1/S1, where the prevalence of damage was lower than this threshold (see Fig. 1); (b) the subjects comprising the current cohort had MCA strokes; therefore, it was not possible to identify “significant” voxel clusters related to strokes in other vascular territories; (c) the parcellation of the White Matter Atlas^63^ does not specify the trajectory of cortico-reticulo-propriospinal fiber tracts and other descending tracts controlling extra pyramidal activity; therefore, we could not assess the possible contribution of damage to these tracts to spasticity and hemiparesis. Second, the VLSM results for the TSRT are based on a lenient criterion, as the results did not survive the FDR correction for multiple comparisons. These results are likely to reflect a trend that may become significant with larger numbers of subjects, although they may also represent a type-1 (false positive) error. Third, we failed to show in the VLSM analysis of MAS results voxel clusters where the existence of damage exerted a statistically significant effect on the test scores. This negative finding probably relates to the fact that patients had to be capable of producing 30° flexion–extension elbow movements with the hemiparetic UL in order to be recruited to the current study. Consequently, the cohort here did not include patients with severe spasticity in elbow flexors (MAS scores ranged between 1 and 2 in all patients). Fourth, the measure of UL paresis used here (FMA-UL) reflects impaired movement in the entire limb, whereas the measure of spasticity (TSRT of BB) is driven from a single muscle. We do not know the extent of anatomical sharing between brain regions involved in regulation of muscle tone in different muscle groups. Moreover, it is common in spastic hemiparesis for different muscles acting on a joint (e.g., extensors and flexors) to exhibit different levels of spasticity. Thus, the relative paucity of brain voxels connected with spasticity in the current study could be affected by the fact that spasticity was quantified here only in one muscle. Fifth, we did not employ a standardized recording of surface EMG at rest, to see if the patients who participated in the study were able or not to abolish all neural activation of muscles at willed relaxation. Thus, uncertainty remains as to the possible contribution of spastic dystonia to the findings.

## Conclusions

The current study sheds new light on differences and similarities in the functional neuroanatomy of UL paresis (FMA-UL) and spasticity (TSRT of spastic elbow flexors). We show that following stroke, hemiparesis and spasticity of the UL are affected differently by lesion topography, with minor anatomical overlapping. The differences, along with the small voxel overlapping, are likely to underlie the complex relationships (comorbidity and divergence) between the manifestation of paresis and spasticity in the upper motor neuron syndrome.

## Supplementary Information

Supplementary Table S1.

## Data Availability

The datasets generated during and/or analyzed during the current study are available from the corresponding author on reasonable request.
