# Supplementary material for: Shared and distinct voxel-based lesion-symptom mappings for spasticity and impaired movement in the hemiparetic upper limb
Source: Sci Rep. 2022 Jun 17;12:10169. doi: 10.1038/s41598-022-14359-8 (PMC9206020; doi:10.1038/s41598-022-14359-8)
Supplement: Supplementary file 1 — Supplementary Table S1. [file 41598_2022_14359_MOESM1_ESM.docx]

**Table S1:**

Title: Demographic and clinical data of individual patients.

| Subject | Sex | Age | Dominance | Lesion side and  vascular territory | Lesion type | Lesion volume (cc) | TAO (months) | MAS | FMA | TSRT |
| --- | --- | --- | --- | --- | --- | --- | --- | --- | --- | --- |
| 101 | M | 46 | R | L MCA | I | 0.36 | 1 | 1.5 | 46 | 93.68 |
| 102 | F | 59 | R | L MCA | I | 2.01 | 1.4 | 1 | 26 | 120.49 |
| 103 | M | 77 | Ambi | L MCA, PCA | I | 182.27 | 2.6 | 1 | 26 | 81.84 |
| 104 | F | 62 | R | L MCA | I | 5.23 | 0.9 | 1.5 | 39 | 128.86 |
| 105 | F | 57 | R | L MCA | I | 7.27 | 1.3 | 1 | 46 | 111.00 |
| 106 | M | 46 | R | L MCA | I | 5.23 | 1.9 | 1.5 | 25 | 102.32 |
| 107 | M | 47 | R | L MCA | H | 11.61 | 2 | 1.5 | 22 | 73.20 |
| 108 | M | 54 | R | L MCA | I | 4.00 | 2.4 | 1.5 | 14 | 128.91 |
| 109 | F | 59 | R | L MCA | I | 5.42 | 1.5 | 1.5 | 52 | 79.50 |
| 110 | F | 51 | R | L MCA | I | 3.35 | 4.3 | 1.5 | 18 | 69.06 |
| 111 | M | 66 | R | L MCA | H | 29.65 | 2.4 | 1.5 | 37 | 129.80 |
| 112 | F | 58 | R | L MCA | I | 5.00 | 2.6 | 1.5 | 30 | 106.17 |
| 113 | M | 54 | R | L MCA, PCA | I | 27.80 | 0.8 | 1.5 | 34 | 162.70 |
| 114 | M | 71 | R | L MCA | H | 15.68 | 1.6 | 1.5 | 46 | 120.56 |
| 115 | M | 48 | R | L MCA | I | 82.33 | 3.1 | 1.5 | 45 | 105.00 |
| 116 | M | 46 | Ambi | L MCA | I | 111.95 | 2.4 | 2 | 14 | 94.54 |
| 117 | F | 74 | R | L MCA | I | 9.38 | 5.9 | 2 | 30 | 119.68 |
| 118 | M | 66 | R | L MCA | I | 5.76 | 3.1 | 1 | 39 | 83.05 |
| 119 | M | 38 | R | L MCA | I | 1.7 | 2.4 | 2 | 51 | 130.57 |
| 120 | M | 74 | Ambi | L MCA, ACA | I | 150.7 | 2.1 | 1.5 | 30 | 100.35 |
| 121 | M | 58 | R | L MCA | I | 0.41 | 2.1 | 1 | 17 | 114.39 |
| 201 | M | 62 | R | R MCA | I | 1.65 | 0.8 | 1 | 52 | 93.68 |
| 202 | F | 71 | R | R MCA | I | 1.36 | 0.8 | 1 | 45 | 161.07 |
| 203 | F | 48 | R | R MCA | I | 24.78 | 0.8 | 1.5 | 26 | 159.96 |
| 204 | F | 72 | R | R MCA | I | 12.55 | 3.9 | 1.5 | 17 | 102.24 |
| 205 | M | 58 | R | R MCA | I | 12.32 | 1.9 | 1.5 | 36 | 85.28 |
| 206 | F | 53 | R | R MCA | I | 5.10 | 2.9 | 1.5 | 22 | 124.62 |
| 207 | M | 38 | R | R MCA | I | 14.89 | 1.9 | 2 | 37 | 129.36 |
| 208 | F | 40 | R | R MCA | H | 127.19 | 1.9 | 1.5 | 33 | 61.72 |
| 209 | F | 50 | R | R MCA, PCA | H | 8.2 | 1.3 | 1 | 38 | 103.01 |
| 210 | M | 53 | R | R MCA | H | 32.81 | 5 | 1.5 | 33 | 91.99 |
| 211 | F | 61 | R | R MCA | I | 2.85 | 1.7 | 1.5 | 17 | 133.30 |
| 212 | M | 41 | R | R MCA | I > H | 32.62 | 3 | 1.5 | 22 | 139.55 |
| 213 | M | 57 | L | R MCA | I | 5.16 | 0.6 | 1 | 36 | 69.62 |
| 214 | M | 56 | R | R MCA | I | 6.72 | 1.5 | 1.5 | 49 | 121.61 |
| 215 | F | 68 | R | R MCA | I | 88.83 | 4.8 | 1.5 | 36 | 74.64 |
| 216 | M | 46 | R | R MCA | I | 65.08 | 3 | 2 | 38 | 88.60 |
| 217 | M | 45 | R | R MCA | H | 62.73 | 3.5 | 1.5 | 14 | 94.12 |
| 218 | M | 41 | R | R MCA | H | 78.89 | 3.1 | 1.5 | 16 | 93.54 |
| 219 | M | 42 | R | R MCA | H | 37.44 | 0.7 | 1.5 | 16 | 134.92 |
| 220 | F | 51 | R | R MCA | I | 11.87 | 1.2 | 1.5 | 54 | 86.67 |

Legend: Dominance L = left, R = right, Ambi = ambidextrous; FMA = Fugl-Meyer assessment scale of the upper extremity; Lesion side L = left, R = right; MAS = Modified Ashworth Scale; Sex M = male, F = female; Stroke type H = hemorrhagic, I = ischemic, I > H = ischemic with hemorrhagic transformation; TAO = time after stroke onset (months); TSRT = tonic stretch reflex threshold; Vascular territory ACA/MCA/PCA = anterior/middle/posterior cerebral artery.
